# Immune responses in multiple hosts to Nucleocapsid Protein (NP) of Crimean-Congo Hemorrhagic Fever Virus (CCHFV)

**DOI:** 10.1371/journal.pntd.0009973

**Published:** 2021-12-01

**Authors:** Elif Karaaslan, Nesibe Selma Çetin, Merve Kalkan-Yazıcı, Sevde Hasanoğlu, Faruk Karakeçili, Aykut Özdarendeli, Ahmet Kalkan, Ali Osman Kılıç, Mehmet Ziya Doymaz

**Affiliations:** 1 Beykoz Institute of Life Sciences and Biotechnology, Bezmialem Vakıf University, Istanbul, Turkey; 2 Department of Medical Microbiology, Faculty of Medicine, Bezmialem Vakıf University, Istanbul, Turkey; 3 Department of Infectious Diseases and Clinical Microbiology, Erzincan University School of Medicine, Erzincan, Turkey; 4 Erciyes University Vectors and Vector Borne Diseases Implementation and Research Center, Kayseri, Turkey; Department of Microbiology, Erciyes University School of Medicine, Kayseri, Turkey; 5 Department of Medical Microbiology, Faculty of Medicine, Erciyes University, Kayseri, Turkey; 6 Department of Infectious Diseases and Clinical Microbiology, Medical Faculty, Karadeniz Technical University, Trabzon, Turkey; 7 Department of Medical Microbiology, Faculty of Medicine, Karadeniz Technical University, Trabzon, Turkey; University of Texas Medical Branch, UNITED STATES

## Abstract

In 2019, the World Health Organization declared 3 billion to be at risk of developing Crimean Congo Hemorrhagic Fever (CCHF). The causative agent of this deadly infection is CCHFV. The data related to the biology and immunology of CCHFV are rather scarce. Due to its indispensable roles in the viral life cycle, NP becomes a logical target for detailed viral immunology studies. In this study, humoral immunity to NP was investigated in CCHF survivors, as well as in immunized mice and rabbits. Abundant antibody response against NP was demonstrated both during natural infection in humans and following experimental immunizations in mice and rabbits. Also, cellular immune responses to recombinant NP (rNP) was detected in multispecies. This study represents the most comprehensive investigation on NP as an inducer of both humoral and cellular immunity in multiple hosts and proves that rNP is an excellent candidate warranting further immunological studies specifically on vaccine investigations.

## Author summary

Crimean Congo Hemorrhagic Fever Virus (CCHFV) is the most lethal human pathogen of medical importance after the dengue virus among arboviruses. The increasing geographic spread of *Hyalomma* ticks, which are responsible for viral transmission widespread, threatens billions of people. WHO currently declares the field of research on CCHFV as the second most urgently needed areas of investigations on emerging pathogens. About 10 to 40% of those infected with the virus lose their life due to the rapidly developing severe clinical manifestations. Pandemic potential and the lack of any approved treatment or vaccine make raise the studies on CCHFV as critical. The studies on CCHFV are challenging due to the necessities of BSL-4 facilities and the immunological characterization of individual structural proteins will lay the groundwork for the steps to be taken to treat and prevent this emerging disease. As is known from other RNA viruses, nucleoprotein (NP) has crucial roles in the viral life cycle, both in viral replication and transcription and in the formation of the virion structure. So far, detailed and comprehensive immunological characterizations on NP in multiple are not undertaken. Our study was set out to embark such detailed investigation. The strong humoral and cellular immune response to NP demonstrated by this study indicates that NP might be an excellent candidate for future scrutinies on vaccines and diagnostic reagents.

## Introduction

Crimean Congo Hemorrhagic Fever Virus is a tick-borne virus belonging to the *Orthonairovius* genus of the *Nairoviridae* family with a tri-segmented genome consisting of small (S), medium (M), and large (L) segments encoding nucleocapsid protein (NP), glycoprotein precursor (GPC) and an RNA-dependent RNA polymerase (RdRp), respectively. CCHFV also encodes nonstructural protein NSs from the S segment in an ambisense orientation and the other nonstructural protein NSm from the M segment after posttranslational cleavage of GPC. Besides, other proteins such as GP38, GP85, GP160, and a mucin-like domain (MLD) are produced during this cleavage [1].

CCHFV has a wide geographical distribution with continued expansion, causing asymptomatic infections or disease generally with mild flu-like to moderate symptoms [2,3]. But in some cases, the disease can progress to a life-threatening phase resulting in hemorrhages, disseminated intravascular coagulation, multiple organ failure, and shock [4,5]. Also, community-acquired, and nosocomial infections are reported [1,6–8].

World Health Organization has declared CCHF as a public health emergency and announced that 3 billion people are at risk of developing this infection. Accordingly, the case fatality rates might reach up to 40%. Therefore, investigations and research activities for this deadly virus should be considered among the prioritized areas of study [9].

In the last two decades, CCHF cases increased remarkably in south-eastern Europe and countries bordering the Black Sea; Georgia, south-western Russia, Turkey, and Ukraine [10]. Since the first cases have emerged in the Kelkit Valley region of Turkey in 2002, a total of 11.041 confirmed cases, including 528 deaths (4.78%) have been reported between 2002–2018 [11].

Immunological response to CCHFV and immune correlates of protection is complex and not fully understood because of the high risk of contagiousness, the requirement of BSL-4 facilities for studies, and the lack of suitable animal models. Little is known about the details of the cellular and humoral immune response to CCHFV and there are many unanswered aspects of CCHFV immunity and most of what we know is obtained from vaccine studies. A consensus seemed to emerge from these studies is that for adequate protection a dominant Th1 mediated response together with strong antibody immunity is needed [12]. However, there are reports indicating a lack of correlation between neutralizing antibodies and protection [12–14].

In many viruses, nucleocapsid proteins are important components of viral life cycles and CCHFV is no exception. CCHFV NP is an indispensable protein because of its role in viral replication and its structural roles in the virion [1,15,16]. Also, the antigenic similarity of NP between genetically and geographically distinct CCHFV isolates makes NP as a potentially safe and logical target antigen in diagnostics, surveillance efforts, vaccine, and drug development studies [17]. All these features suggest that NP may also have an indispensable role in immunity. NP’s function in innate immunity has been investigated in some studies. For instance, interactions between NP and the elements of innate immunity have been addressed. These results point out NP as an innate immunity regulator through modulation of interferon/cytokine response and apoptosis pathways [18–22].

Research focusing specifically on NP and its role in adaptive immunity to CCHFV is relatively scarce, and the results are controversial. Epitope mapping studies revealed that NP is highly immunogenic and has antigenic epitopes scattered throughout the protein [17,23–26]. These epitopes are recognized by antibodies from viremic animals [23,25] and actively infected or convalescent human sera [17,26]. Data obtained from CCHF survivors demonstrate that T cell response to NP epitopes was in abundance compared to glycoproteins. These data seem to indicate the importance of NP as an antigen for the recovery from infection [27]. Some argued that NP-specific antibodies have very little function in viral neutralization and do not necessarily correlate with complete protection [28,29].

In consideration of the data on NP, we set out on a detailed analysis of the immunological features of NP. Therefore, in this report, both humoral and cellular aspects of the immune response to rNP was addressed. We questioned the immunological characteristics of rNP and its role as an inducer of humoral and cellular immunity in multiple species.

## Materials and methods

### Ethics statement

The study protocols of both human and animal experiments have received ethical clearance from the Ethics Committee for Non-Invasive Studies (Approval numbers: 71306642/050-01-04/228 and 54022451–050.05.04) and the Ethics Committee for Animals Studies (Approval number: 213/239) at Bezmialem Vakıf University in Istanbul, Turkey.

### Virus propagation and inactivation

Crimean Congo Hemorrhagic Fever Virus Kelkit06 strain, isolated from a patient’s serum sample, used in the study [30]. All virological assays with live viruses were performed in a BSL-3 enhanced facility of Erciyes University Vaccine Research and Development Application and Research Center, Turkey. The virus stocks of CCHFV Kelkit06 for immunizations of mice and rabbits were propagated in Vero E6 cells. Vero E6 cells were infected with CCHFV at MOI of 0.01 and the supernatants were harvested at three days post-infection (dpi). The viral particles were purified by using sucrose gradient ultracentrifugation, inactivated by incubation with 0.05% formaldehyde for three hours at room temperature. Then, the formaldehyde was removed by dialysis with phosphate-buffered saline (PBS) using a 20K MWCO dialysis cassette (Millipore, Bedford, MA). The amount of protein in immunizations with inactivated virus was measured with the Lowry Protein Assay Kit (Thermo Fisher Scientific, Waltham, USA).

### Plasmid constructions

CCHFV Kelkit06 NP (1449 bp) retrieved from GenBank (Accession no. GQ337053) was optimized for bacterial expression, synthesized, and cloned into pUC19 by GenScript USA Inc. (Nanjing, Jiangning, China) and then, propagated in DH5α *Escherichia coli* cells. For the expression, CCHFV NP was subcloned into pET28b (Addgene, Cambridge, USA) by using *Xho*I (Sigma-Aldrich, Taufkirchen, Germany) and *Nco*I (New England Biolabs, Massachusetts, USA) restriction enzymes and transformed into One Shot BL21(DE3) Chemically Competent *E*. *coli* cells (Thermo Fisher Scientific, Waltham, USA). After transformation, positive transformants were selected with kanamycin and analyzed by sequencing.

### His-tagged protein expression and purification

An overnight culture with LB containing 50μg/ml kanamycin was prepared for each positive transformants. When OD_600_ 1–2 was reached, overnight culture was diluted 1:50 in LB containing 1% (w/v) glucose and incubated at 37°C with constant stirring at 220 rpm to reach OD_600_ 0.4–0.6. Then, the cells were induced with 1 mM isopropyl β-d-thiogalactopyranoside, IPTG (Sigma-Aldrich, Taufkirchen, Germany) and cultured at 37°C on a rotary incubator shaker at 220 rpm for 3 hours. To analyze the solubility of the expressed protein, 1 ml culture was lysed with lysozyme containing native lysis buffer (0,2 mM Lysozyme, 1% Triton X-100, 10% Glycerol, pH 7,4). For the purification of the expressed proteins under denaturing conditions, the cells were harvested by centrifugation at 5000*g* for 10 minutes at 4°C and washed with PBS. Before purification, cells were incubated on ice for 30 minutes; resuspended in Lysis Buffer (8 M urea, 100 mM NaH_2_PO_4_ and 10 mM Tris-Cl, pH 8.5); sonicated for 3 times 10 s bursts (Sonopuls HD 2070, BANDELIN electronic GmbH & Co. KG, Berlin, Germany); freeze-thawed 3 times; incubated at room temperature with agitation for 1 hour; centrifuged at 10.000*g* for 10 minutes. Clear lysates were collected while pellets were stored at -20°C for SDS-PAGE analysis. Purification of rNP was performed on ÄKTA pure chromatography system (GE Healthcare Life Sciences, Glattbrugg, Switzerland) through HisTrap Excel columns (GE Healthcare Life Sciences) according to the manufacturer’s instructions under denaturing conditions. Before applying cleared lysates to the column, samples were centrifuged at 50.000g for 30 minutes to prevent any clogging during chromatography. The fractions were analyzed by 12% SDS-PAGE and protein concentrations were determined by both Bradford Protein Assay Kit (Thermo Fisher Scientific, Waltham, USA), according to manufacturer’s instructions and absorbance values measured at 280nm on NanoDrop 8000 Spectrophotometer (Thermo Fisher Scientific, Waltham, USA). To re-fold pure denatured proteins, dialysis was performed at room temperature against PBS by using SnakeSkin Dialysis Tubing (Thermo Fisher Scientific, Waltham, USA).

### Collection of human, mouse and rabbit blood and serum samples

#### Human blood and serum samples

For the enzyme immunoassays, 28 CCHFV antibody-positive human sera that have been confirmed by the reference laboratories of the Public Health Institution at Ministry of Health, by the polymerase chain reaction, have been used. Serum samples of CCHFV survivors were obtained from blood collected at routine controls of patients following discharge. Stored serum samples were also tested with VectoCrimea-CHF-IgG kit (Novosibirsk, Russia) to detect CCHFV-specific IgG antibodies. Twenty-three CCHFV antibody-negative human sera obtained from Bezmialem Vakif University from the patients that admitted for routine examinations that do not have a disease that can interfere with EIA results like infectious diseases, cancer, diabetes, hypertension, immunodeficiency, and autoimmune diseases. For the lymphoproliferation assay (LPA), follow-up blood samples from three patients admitted to Erzincan Binali Yıldırım University that had previous CCHFV infection were used. The presence of IgG antibodies at the follow-up blood sample was the sole criteria for inclusion in the study and the previously mentioned exclusion criteria were also applied. Whole blood collected to heparin tubes on the day of follow-up used immediately for the LPA.

#### Mouse blood and serum samples

Six to eight weeks old male Balb/C mice were used for both rNP and inactivated CCHFV (iCCHFV) immunizations. Each group had five mice including negative control and positive control groups. Blood samples were collected from the facial vein two-weeks following each immunization. Following the collection of blood, samples were incubated at room temperature for 30 minutes to allow blood to clot and sera were separated after centrifugation at 1.000g for 10 minutes. The aliquots of separated serum samples were stored at -20°C until further use. For the LPA, mice were euthanized with cervical dislocation under isoflurane anesthesia and spleen and lymph nodes collected under aseptic conditions.

#### Rabbit blood and serum samples

Eight to ten week old two New Zealand White rabbits were immunized with iCCHFV Kelkit06 strain. For the EIA, 500μl of blood collected from marginal ear vein and sera were separated as described above. For the LPA assay, 5ml of blood collected from central ear artery in heparin tubes and used at the same day of blood collection.

### Immunizations

To assess the anti-NP response in rabbits, Two New Zealand White rabbits were immunized with iCCHFV Kelkit06 strain. For the first immunization, 60μg/ml iCCHFV Kelkit06 strain emulsified in Freund’s Complete Adjuvant (CFA) (Sigma-Aldrich, Taufkirchen, Germany). For the booster immunizations, same amount of iCCHFV Kelkit06 strain emulsified in Incomplete Freund Adjuvant (IFA) (Sigma-Aldrich, Taufkirchen, Germany). Immunizations performed by subcutaneous route from the neck into four different sites with 6μg iCCHFV Kelkit06 strain in 200μl per site (24μg in 800μl in total) and boosted four times at monthly intervals with the same amount of iCCHFV Kelkit06 strain. Blood was collected from rabbits every 15 days, and serum samples were analyzed by enzyme immune assay. Naïve (unprimed) rabbit serum was used as a negative control.

Six to eight weeks old Balb/C mice obtained from Bezmialem Vakif University Research Animals Laboratory were inoculated subcutaneously into the shoulder blade or flanks with 100μl of 1 mg/ml CCHFV rNP emulsified in CFA at a final vaccination dose of 100μg rNP. With two week intervals, mice were boosted two times with the same dose of antigen emulsified in IFA by the same route. Bovine serum albumin (BSA) (Sigma-Aldrich, Taufkirchen, Germany) emulsified in relevant adjuvants was used in positive control groups and 100μg BSA was given as final vaccination dose. Mice in negative control groups were mock immunized with PBS emulsified in relevant adjuvants. Immunizations performed with the same schedule as described above.

To determine rNP-specific humoral immune response in mice immunized with iCCHFV, six to eight-week-old Balb/C mice were immunized intraperitoneally with 10 μg iCCHFV in 200μl in aluminum hydroxide (Sigma-Aldrich, Taufkirchen, Germany) with three week intervals. Each mouse in the control group was injected with aluminum hydroxide without antigen, following the dose and method applied in the experimental group.

### SDS PAGE and western blotting

The protein samples were diluted 1:1 in Laemmli buffer (4% (w/v) SDS, 10% (v/v) β-mercaptoethanol, 20% (v/v) glycerol, 0,004% (w/v) bromophenol blue, 0,125 M Tris-HCI) and heated to 95°C for 10 min. Protein samples were separated on 12% polyacrylamide gel by SDS-PAGE and stained with Coomassie brilliant blue G-250 (Sigma-Aldrich, Taufkirchen, Germany) for the detection of target protein. For the detection of proteins by Western Blotting, the protein samples were separated as described above and transferred to a nitrocellulose membrane in transfer buffer (24 mM Tris, 192 mM glycine and 20% (v/v) methanol) at 25 V for 7 minutes on semi-dry blotter (Trans-Blot Turbo, Bio-Rad, California, USA). The membrane was blocked with TBST (10 mM Tris pH 7.4, 0.9% (w/v) NaCl, and 0.05% (v/v) Tween-20) containing 5% (w/v) skim milk (Sigma-Aldrich, Taufkirchen, Germany) for two hours at room temperature and incubated with primary antibody overnight at 4°C. The human, mouse, or rabbit sera were used as primary antibodies in 1:1.000, 1:100 and 1:10 dilutions, respectively. Also, monoclonal antibody 2D3 (is an anti-NP monoclonal antibody generated in Dr. Doymaz’s lab) was tested against rNP. The membrane was washed five times with TBST, then incubated with goat anti-human HRP (ab81202, Abcam, Cambridge, UK), goat anti-mouse IgG HRP (sc2031, Santa Cruz Biotechnology, California, USA), goat polyclonal 6X His tag-HRP (ab1269, Abcam, Cambridge, UK) or goat anti-rabbit IgG HRP (Santa Cruz Biotechnology, California, USA) at room temperature for one hour. Finally, the membrane was washed five times and processed for chemiluminescence detection (WesternBright Sirius Chemiluminescent Detection Kit, Advansta, California, USA), according to the manufacturer’s instructions. Membranes were visualized by using Fusion FX Solo imaging system (Vilber, Collégien, France).

### Delayed-type hypersensitivity (DTH)

One week after the last immunization, 50 μl of 1 mg/ml rNP (50 μg in final) in PBS was injected in the right hind footpad of rNP-immunized Balb/C mice and the same volume of PBS into their left footpad. For the positive control group, same amount of BSA in PBS was injected in the right hind footpad of mice immunized with BSA. Footpad thickness was measured in 24-hour intervals for three days following challenge with External Electronic Caliper Gauge (Swiss Precision Instruments, Melville, NY 11747, USA).

### PBMC isolation

Peripheral blood mononuclear cells (PBMCs) were isolated from heparinized peripheral blood collected aseptically from 3 CCHFV survivors and rabbits immunized with iCCHFV. Three ml of blood samples were layered 1:1 onto Histopaque-1077 (Sigma-Aldrich, Taufkirchen, Germany). Following density gradient centrifugation for 30 min at 400 *g* cells were washed twice. Isolated PBMCs were diluted to 1.5x10^6^/ml in RPMI 1640 (Sigma-Aldrich), supplemented with 10% (v/v) FBS (Gibco, Thermo Fisher Scientific, Waltham, USA) and 1% (v/v) penicillin/streptomycin (PES) (Thermo Fisher Scientific, Waltham, USA). The viability and number of cells was assessed by trypan blue (Sigma-Aldrich, Taufkirchen, Germany).

### Isolation of murine splenocyte and lymphocyte

One week after the last immunization, lymph nodes and spleen of rNP immunized mice were aseptically removed to obtain a single-cell suspension. Lymph nodes and spleen were homogenized between the frosted ends of the sterile slides, separately. The cells were washed twice with Dulbecco’s phosphate-buffered saline (dPBS) (Gibco, Thermo Fisher Scientific, Waltham, USA) and spun at 400*g* for five minutes. The homogenized spleen cell pellet was resuspended in ACK buffer (150 mM NH_4_Cl, 10 mM KHCO_3,_ and 0.1 mM Na_2_EDTA, pH 7.4) and incubated for two minutes at room temperature to lyse red blood cells. Subsequently, 10 ml dPBS was added and spun at 400*g* for 4 minutes. The supernatants were discarded and splenocytes and lymphocytes were resuspended in RPMI 1640 supplemented with 10% (v/v) FBS and 1% (v/v) PES. The cells were pooled after the determination of the cell number and viability assessed by trypan blue.

### Lymphocyte proliferation assay (LPA)

Proliferation assays were carried out either with PBMCs from human or rabbit blood or with mouse lymphocytes and splenocytes as combined. Lymphocyte proliferation assays were performed by a commercial 5-Bromo-2’-deoxyuridine (BrdU) colorimetric kit (Roche Diagnostics, Indianapolis, USA). Briefly, for rabbit and human assays 1.5x10^5^ PBMC/well or for mice assays 10^5^ lymphocytes/well combined with 0.5x10^5^ splenocytes/well were seeded on 96 well flat bottom tissue culture plate (TPP, Sigma-Aldrich, Taufkirchen, Germany). The plates were prepared to contain different concentrations of rNP (1, 0.5 and 0.1μg/ml) and concanavalin A (1μg/ml, as a positive control) at the time zero. The assays were performed in triplicate with RPMI 1640 supplemented with 10% (v/v) FBS and 1% (v/v) PES as a culture media. After two days of incubation, cells were labeled for 24 hours with 10μM BrdU labeling solution. The ratio of BrdU incorporation into the cells measured according to the manufacturer’s instructions and the results are presented as percent response for the antigen. The assays performed in triplicate. The percent response is calculated as follows: [(mean OD of the antigen-stimulated group–mean OD of the negative control group)/mean OD of the negative control group] x100.

### Cytokine profiling

The mice lymphocytes and splenocytes were stimulated with rNP as described at LPA and culture supernatants were harvested and analyzed by the Mouse Multi-Analyte ELISA Array (Qiagen, Germantown, MD) containing interleukin (IL)-1B, IL-4, IL-6, IL-10, IL-12, IL-17A, IFN-γ, G-CSF, and GM-CSF according to the manufacturer’s protocol. The data was obtained by measurement of absorbance at 450nm by a microplate reader. Absorbance values more than two times the negative control absorbance values are interpreted as positive samples according to the manufacturer’s instructions.

### Enzyme immunoassay (EIA)

Microplates (Immulon 2 HB, Invitrogen, Waltham, USA) were coated overnight at 4°C with rNP diluted in carbonate-bicarbonate buffer (pH 9.6) at a final concentration of 1μg/well. Then, the plates were washed four times with PBST (PBS + 0.05% (v/v) Tween 20) and blocked with PBST containing 5% (w/v) skim milk for two hours at room temperature. For human EIA, 28 CCHFV antibody-positive and 23 CCHFV antibody-negative human sera confirmed in the VectoCrimea-CHF-IgG ELISA kit (Vector-Best, Novosibirsk, Russia) were used as primary antibodies in 1/100 dilution. For the detection of antibody titers in inactive CCHFV immunized rabbit and rNP-immunized mice sera 1/10 and 1/100 dilutions used, respectively. Antibody titration EIAs were performed on selected serum samples. The plates were incubated at 37°C for one hour and washed four times with PBST. As secondary antibodies, goat anti-human IgG HRP (Abcam, Cambridge, UK), goat anti-mouse IgG HRP (Thermo, Waltham, USA), and goat anti-rabbit IgG HRP (Santa Cruz Biotechnology, Texas, USA) were added to the wells, depending on analytes. Then, the plate was incubated for one more hour at 37°C. The wells were washed three times with PBST and incubated with TMB solution (Abcam, Cambridge, UK) for 20–25 minutes at room temperature in the dark. The reaction was stopped by adding 2N H_2_SO_4_ to the wells and absorbance of samples measured in iMark microplate reader (Bio-Rad, California, USA) at 450 nm. All washing steps performed using Wellwash Versa Microplate Washer (Thermo Fisher Scientific, Waltham, USA). Data have shown here represent the mean of three independent experiments performed in duplicates. Also, serum samples were serially diluted to detect endpoint IgG levels in human, rabbit and mice sera and EIA performed as described above.

### Statistical analysis

The statistical analyses were made by the Prism 6 for OS X Version 6.0.1 (GraphPad 232 Software, San Diego, CA) program. Results from the DTH assay were expressed as mean ±SEM of the difference between the thickness of the left and right hind footpads. Two-way ANOVA analysis and Bonferroni post-tests were used for statistical analysis. The cut-off value for Antibody Titration EIA was calculated by the given formula: the mean absorbance of each CCHFV negative samples + 2 SD. Significance between grouped data was determined by Mann–Whitney U tests or a two-tailed Welch’s t-test. Significance for all comparisons was accepted at *p*<0.05 value (**p<0.01, ***p<0.001, ****p< 0.0001).

## Results

### Production of recombinant NPs of CCHFV

**Fig 1:**
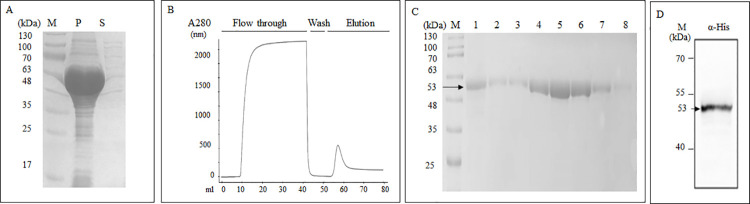
Expression and purification of CCHFV rNP by Ni-NTA affinity chromatography. (A) Analysis of the rNP solubility in fractions of cell extracts on Coomassie stained 12% Tris-Glycine SDS-PAGE gel after expression in BL21 (DE3) with 1 mM IPTG induction for three hours. M: marker (Cat. #24052, Intron Biotechnology), P: pellet, S: supernatant samples obtained after lysis of bacteria under native conditions. (B) Chromatogram of 6xHis-tagged CCHFV rNP monitored by UV 280 nm absorbance with ÄKTA pure chromatography through HisTrap Excel columns. (C) Analysis of elution fractions obtained from after Ni-NTA Column Chromatography on Coomassie stained 12% Tris-Glycine SDS-PAGE gel. M: marker (Cat. #24052, Intron Biotechnology), 1–8: Elution fractions. D) Western blot analysis of purified rNP using goat polyclonal anti His tag-HRP at 1:1000 dilution.

The optimized CCHFV NP gene was cloned into pET28b by directional cloning and expressed with a C-terminal polyhistidine tag in a prokaryotic expression system. After expression, rNP remained in the insoluble fraction even though bacterial cells were lysed with lysozyme containing native lysis buffer (Fig 1A). Therefore, it was purified by affinity chromatography under denaturing conditions (Fig 1B) and refolded in PBS by dialysis. The purification efficiency and protein quantity in each elution fraction were evaluated by SDS-PAGE analysis. In this analysis, the band corresponding to ~ 53 kDa shows that the produced protein is in the expected size and purity (Fig 1C). Also, Western blot analysis with the anti-His Tag antibody has once again confirmed these findings (Fig 1D).

### Bacterially expressed NP has the authenticity of CCHFV NP

**Fig 2:**
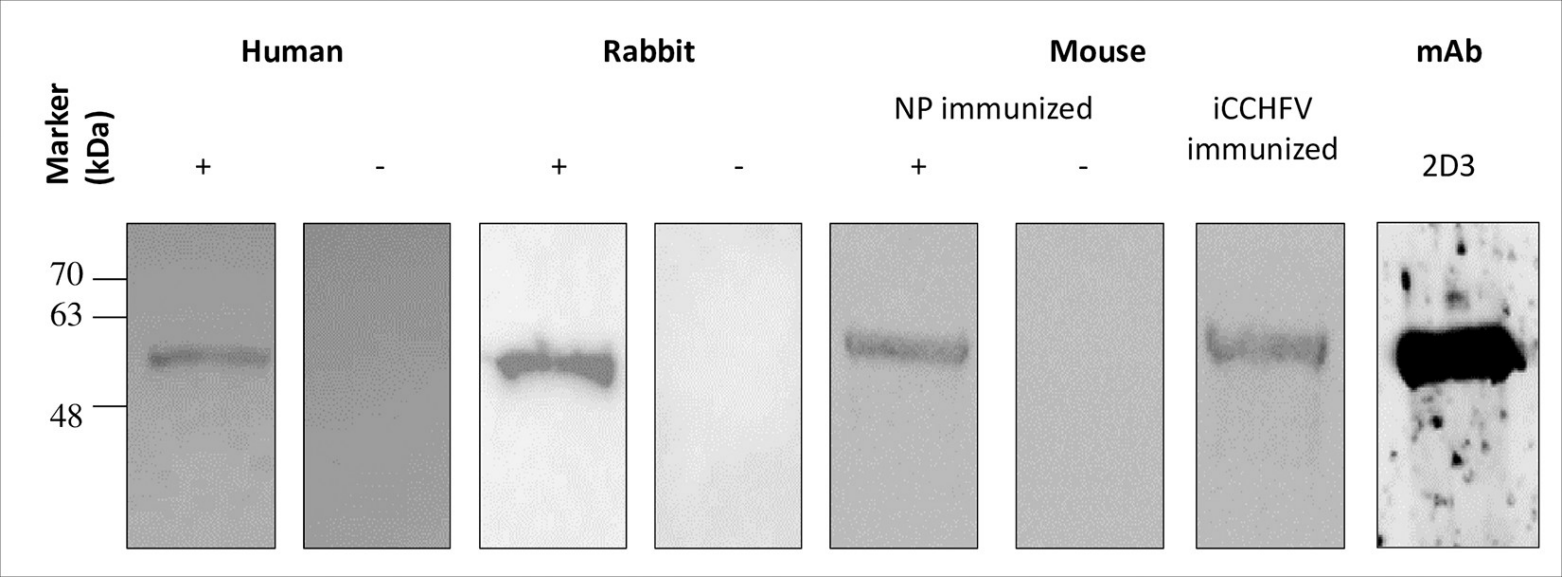
Analysis of the authenticity of rNP. Purified rNP ran on 12% polyacrylamide gel by SDS-PAGE and transferred on nitrocellulose membranes. Western blot analysis performed with serum samples from different species that have anti-CCHFV antibodies (CCHFV survivors and iCCHFV immunized rabbits) or anti-NP antibodies (rNP immunized mice samples and anti-rNP mAb; 2D3). Except for rabbit serum (1:10), all primary antibodies were prepared at 1:1.000 final dilution in blocking buffer. (+): CCHFV infected or immunized/rNP immunized; (-): mock immunized rabbit or mice, or healthy human sera.

To further utilize bacterially expressed rNP as a surrogate of CCHFV NP, the authentic antigenicity of the produced protein was established. The authenticity of rNP has been validated by different methods in multi-species. First, reactions of rNP with antibodies raised in rNP-immunized mice, convalescent human sera, and iCCHFV-immunized rabbit and mice revealed that recombinantly expressed NP had authentic antigenicity and therefore it can serve as a surrogate for viral NP antigen of CCHFV (Fig 2).

**Fig 3:**
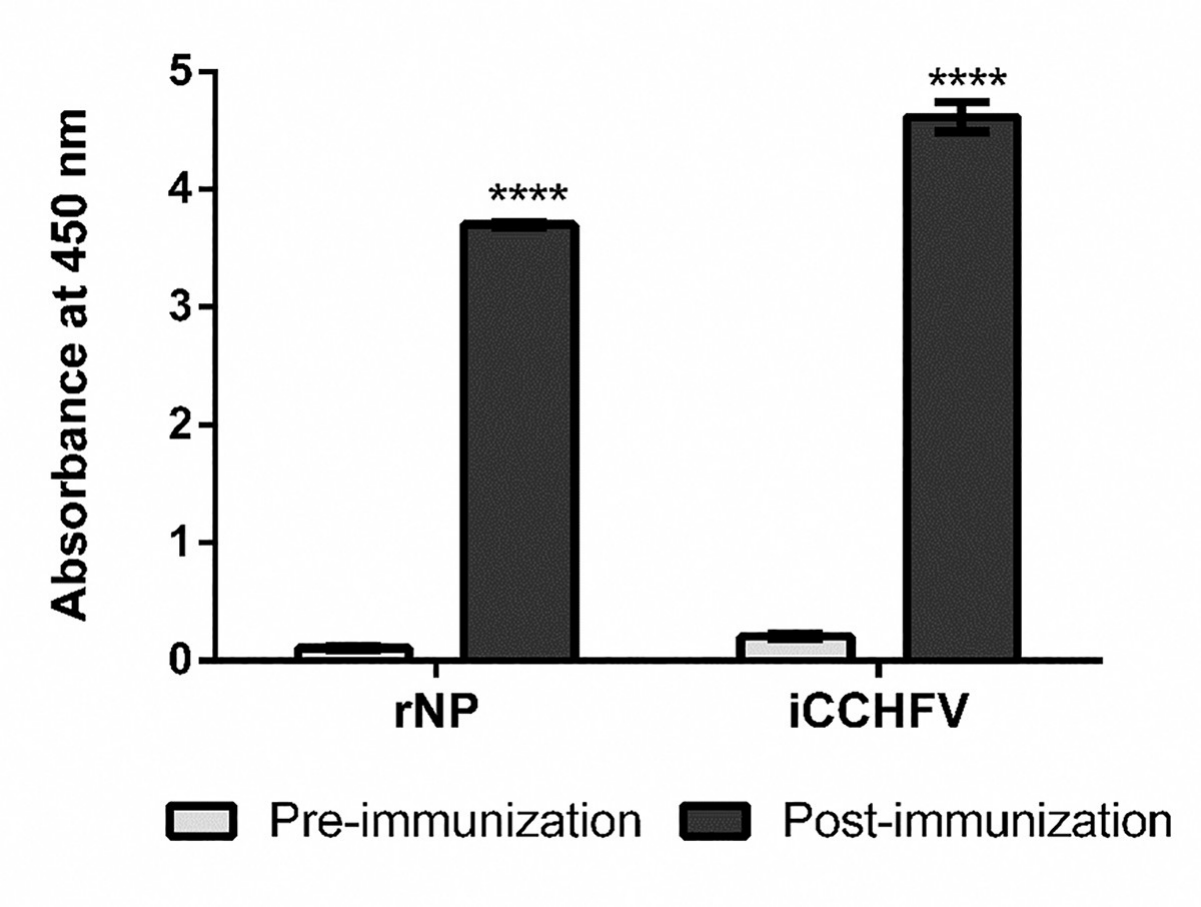
Analysis of antigenic fidelity of recombinant NP by EIA. EIA plates were coated either with rNP (1μg/well) or whole viral antigen (1μg/well) and analyzed using sera, collected from a mouse pre- and post-immunization with inactive CCHFV at 1:1000 final dilution. The NP specific antibodies were detected with HRP-conjugated anti-mouse IgG antibody. Each sample was tested in duplicate and measured at the absorbance value of 450 nm, and the bars represent the mean value of samples. Each data represents mean ± SEM. Pre- and post- immunization data for each group was statistically analyzed separately by Welch’s t-test. **** *p*< 0.0001.

Also, to further confirm the antigenicity of the recombinant protein, rNP was tested in EIA at Erciyes University Vaccine Development Center using sera of a mouse, collected from pre-and post-immunization with inactive CCHFV Kelkit06 strain. Here, rNP and whole CCHFV were used as solid antigens to compare the binding capability of antibodies generated against CCHFV. The results of this test once again demonstrated antigenic fidelity of rNP and the ability of the rNP to induce a high titer antibody response in mice (Fig 3).

**Fig 4:**
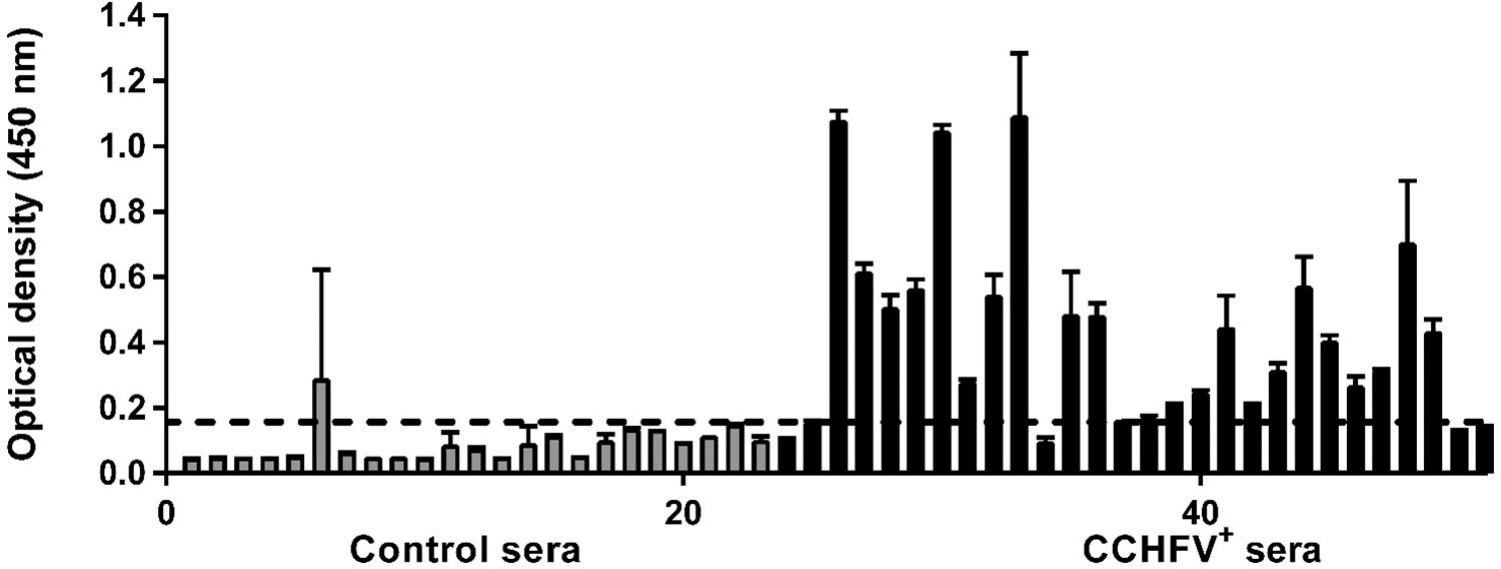
Evaluation of NP-specific antibody response using CCHF convalescent sera. A serum panel consisting of 28 CCHFV-IgG positive and 23 CCHFV-IgG-negative serum samples were analyzed by in-house CCHFV rNP based IgG EIA. The plates were coated with rNP (1μg/well), serum samples were used at 1:100 final dilution and detected with HRP-conjugated anti-mouse IgG antibody. The mean optical density obtained from anti-CCHFV IgG negative human sera samples was 0.070 and standard deviation was 0.030. The difference of optical densities between anti-CCHFV IgG antibody positive and negative sera groups was analyzed by Mann-Whitney U test and found statistically significant. (*p*< 0,0001). The cut-off value was calculated as follows: 2SD + mean absorbance of each CCHFV negative samples. Each data represents mean ± SEM. Each data was calculated by averaging the optical density values obtained from three independent experiments performed in duplicates at an absorbance value of 450 nm. Gray bars represent CCHFV antibody negative, black bars represent CCHFV positive human sera.

To assess the antigenic potential of rNP, next, we tested this antigen with CCHFV antibody-positive serum obtained from survivors. For this purpose, 28 CCHFV specific antibody positive and 23 negative human serum were used, and NP-specific IgG was probed. Of the samples tested, the in-house rNP EIA detected a total of 25 out of 28 CCHFV-specific antibody positive sera as positive and 21 out of 23 CCHFV-specific antibody negative samples as negative. Accordingly, sensitivity and specificity of the in-house CCHFV IgG EIA test were found as 89,2% and 91,3%, respectively (Fig 4). These results indicated that NP is a strong inducer of the humoral immune response during natural CCHFV infection. These data also imply that an rNP-based EIA can solely serve as a good surrogate to commercial diagnostic assays using the whole virus as antigen.

### The CCHFV NP induces humoral immunity in multi-species

**Fig 5:**
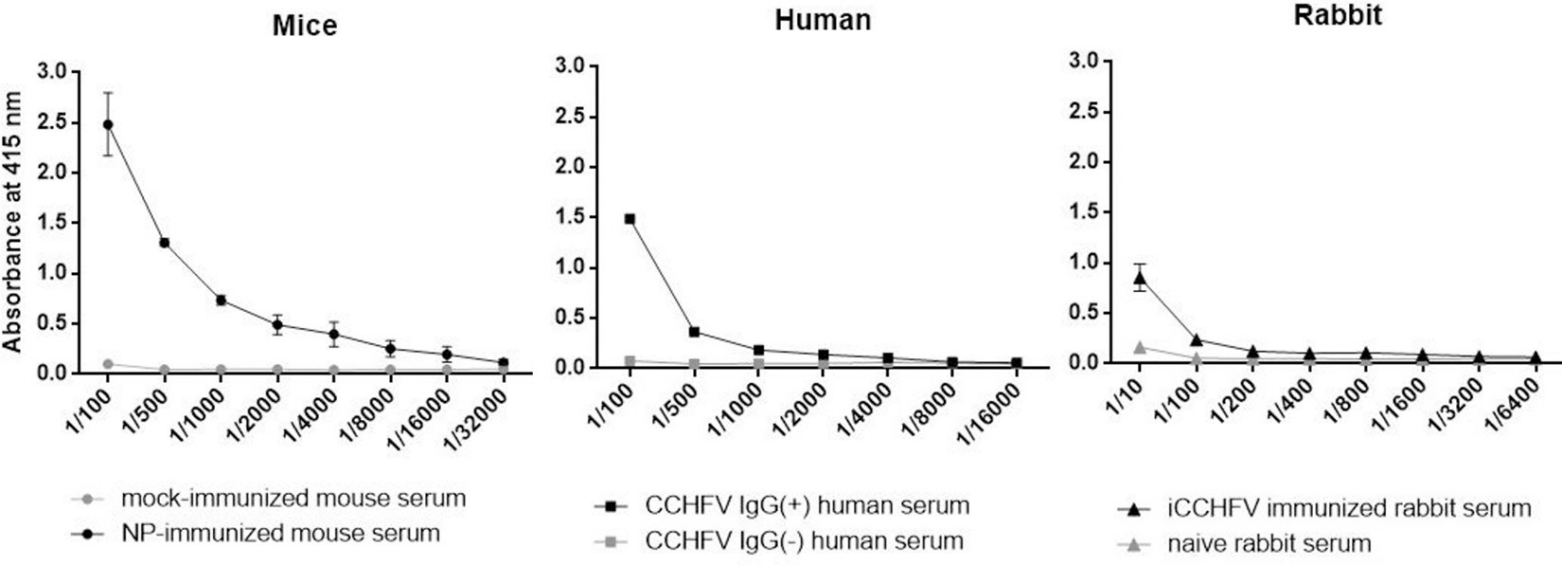
Detection of NP-specific IgG antibodies in multiple hosts. The endpoint NP-specific IgG titers in sera samples from different species were determined with EIA in which rNP (1μg/well) was utilized as a solid phase antigen. Serum samples were serially diluted in a range of 1:100 to 1:32000 for mice sera, 1:100 to 1:16000 for human sera, and 1:10 to 1:6400 for rabbit sera and incubated with the relevant HRP-conjugated secondary antibodies. To detect the endpoint IgG titers for each species, cut-off values were calculated by using the corresponding dilution of the related negative sample. The cut-off values were determined as follows: 2SD + mean absorbance of each negative sample. Each data represents mean ± SEM, calculated by averaging the optical density values measured from three independent experiments performed in duplicates at an absorbance value of 415 nm. iCCHFV: Inactivated CCHFV.

The extent to which humoral immunity induced by NP examined using different mammalian sera by in-house developed EIA test where rNP was used as a solid phase antigen. To show both the titer of the antibody response generated in immunized mice and their ability to detect antibodies from CCHFV immunized mice sera, an in-house EIA test was performed. The EIA test results showed that rNP was able to induce an antibody response that was detectable even at 1:32.000 dilutions (OD415: 0.117, the cut-off value: 0.058) (Fig 5A). Then, with the same method, rNP was also able to capture antibodies in serum samples of CCHFV survivors even at 1:16.000 dilution (OD415: 0.058, the cut-off value: 0.057) (Fig 5B). Also, two New Zealand White rabbits were immunized with iCCHFV Kelkit06 strain. In rabbit sera immunized with iCCHFV, apparent NP-specific antibody response was observed at 1:6.400 dilution of immunized rabbit sera compared to the unprimed animals (OD415: 0.067, the cut-off value: 0.059) (Fig 5C).

### The CCHFV NP induces cell-mediated immunity

**Fig 6:**
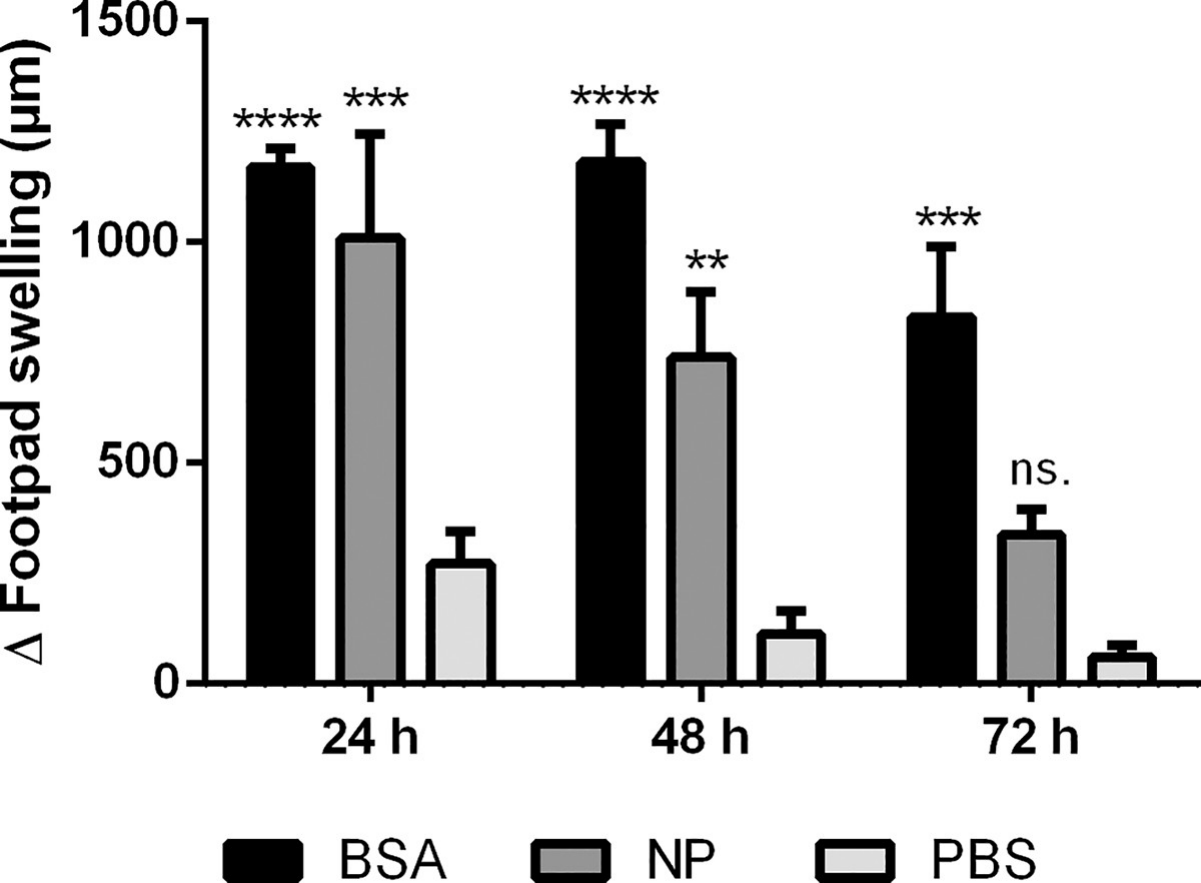
rNP-elicited footpad swelling in mice. Mice were immunized with rNP as test antigen or with BSA and PBS as positive and negative controls, respectively as described. One week after last immunization, DTH reactions in the footpad were induced by a subcutaneous rNP (50 μg in final), BSA (50 μg in final) or PBS challenge. The thickness of footpads was measured before and 24, 48 and 72 hours after challenge by electronic caliper gauge. Each bar represents mean values ± SEM of each immunization group (The data were from 5 mice per group). PBS and BSA were utilized as a negative and positive control, respectively. The differences of mean footpad thickness of mice compared to corresponding negative controls were statistically significant (**, *p* < 0.01; ***, *p*< 0.001; ****, *p*< 0.0001; and ns, not significant). Two-way ANOVA and Bonferroni’s multiple comparison tests were used for statistical analysis between all groups at the same time point. ΔFootpad Swelling: The difference in the thickness of antigen injected and PBS injected footpad, BSA: Bovine serum albumin, NP: nucleoprotein, PBS: Phospate buffered saline.

To determine the effect of CCHFV rNP on Th1 responses *in vivo*, we assessed the delayed-type hypersensitivity responses (DTH) in the mouse model. Both rNP- and mock immunized mice were challenged after the injection of protein in the right hind footpad and an identical volume of PBS in the left hind footpad. The differences between the thickness of the right and left hind footpads resulting from the inflammatory edema was measured at different time points to assess the intensity of the DTH response. As a result of these measurements, the increase in the thickness due to infiltration was measured as 49.4±12.8% (*p* <0.001) at 24 hours compared to the negative control. DTH response persisted with 39±8.2% (*p* <0.01) incrassation measured at 48 hours and decreased to 18.7±3.1% (*p*˃ 0.05) at 72 hours. These results confirm that CCHFV rNP induced a significant DTH in immunized mice compared to control group animals (Fig 6).

The contribution of CCHFV rNP to the T cell-mediated immune response was further evaluated in mice, rabbits, and humans. To that end, lymphocytes from NP-immunized mice combined with splenocytes or PBMCs of rabbits immunized with formalin-inactivated CCHFV Kelkit06 were stimulated with various concentrations of CCHFV rNP. Stimulation with 100ng rNP resulted in 72% more lymphoproliferative response in mouse and 141% in rabbit T-cells compared to non-stimulated control cells. In the case of natural infection, PBMCs from different CCHFV survivors stimulated with rNP to assess antigen-specific lymphoproliferative response, and varying degrees of lymphoproliferation measured in three different survivors, with one being as high as 680% with 500 ng rNP (Table 1). These results suggest that the rNP protein has epitopes that can be recognized by T cells from humans, mice, and rabbits.

**Table 1:** The percentage of lymphoproliferative response^a^ induced by CCHFV rNP antigens at different doses in multiple species.

Group		Stimulant	Antigen Amount For Stimulation in μg	Lymphoproliferation	
				SE	% response^b^
**Mice**		**NP**	1	0.025	50
			0,5	0.038	64
			0,1	0.112	72
			0	0.004	0
		**ConA**	1	0.068	106
**Rabbit**		**NP**	0,5	0.038	10
			0,1	0.112	141
			0	0.04	0
		**ConA**	1	0.068	94
**Human**	**Patient 1**	**NP**	1	0.021	435
			0,5	0.021	680
			0,1	0.001	196
			0	0.011	0
		**ConA**	1	0.094	8978
	**Patient 2**	**NP**	1	0.021	72
			0,5	0.021	74
			0	0.011	0
		**ConA**	1	0.093	5460
	**Patient 3**	**NP**	1	0.014	43
			0,5	0.005	24
			0,1	0.020	1.9
			0	0.012	0
		**ConA**	1	0.029	757

^a^ To detect the lymphoproliferative response in mice and rabbits, lymphocytes and splenocytes of mice immunized with rNP and PBMCs of rabbits immunized with iCCHFV were harvested one week after last immunization. To detect lymphoproliferative response in human, PBMCs were isolated from CCHFV survivors. A total of 1.5x10^5^ cells/well (lymphocytes and splenocytes combined in 1:0,5 ratio for mice and PBMCs for rabbits and humans) were stimulated with varying concentrations of rNP (1, 0.5 and 0.1μg/ml) and with concanavalin A (1μg/ml) as positive control. After two days of incubation, cells were labeled for 24 hours with 10μM BrdU labeling solution. The increase in the cell number was detected by measuring the BrdU-labeled DNA. The reaction between anti-BrdU antibody and incorporated BrdU measured colorimetrically at 450 nm.

^b^ Percent response is calculated as follows: [(mean OD of the antigen-stimulated group–mean OD of the negative control group)/mean OD of the negative control group] x100. SE: standard error of the mean, OD: optical density, con A: concanavalin A.

**Fig 7:**
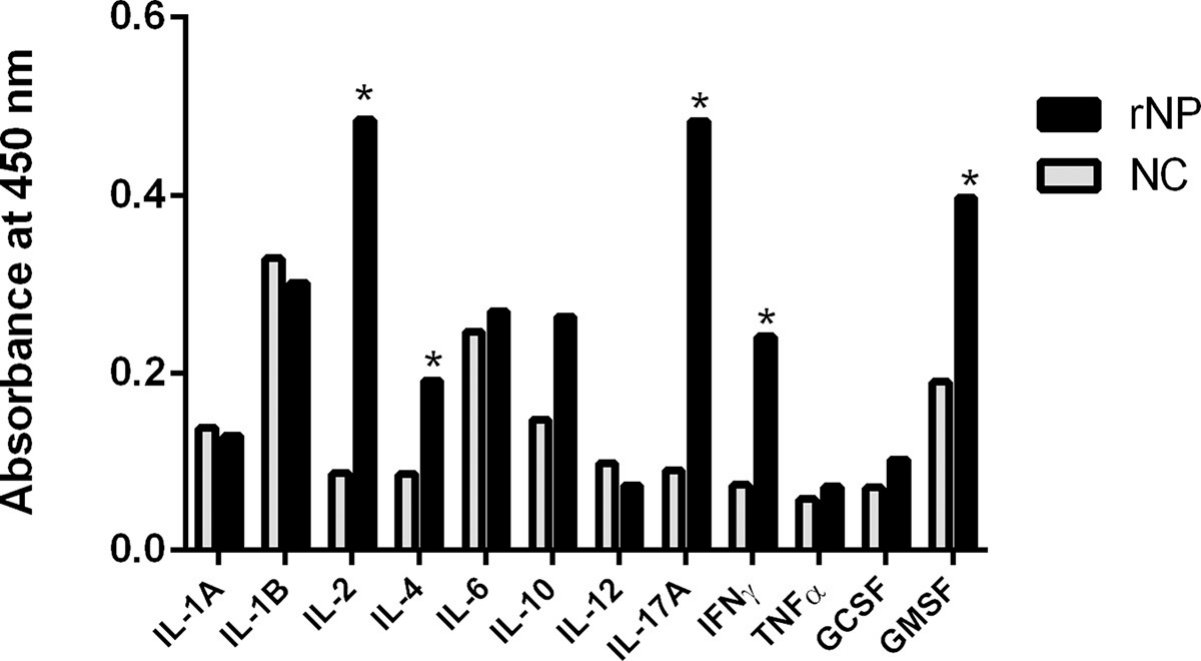
Measurement of cytokines released into CCHFV rNP stimulated mice lymphocytes medium. The media of combined lymphocyte and splenocytes cultures of mice containing secreted cytokines were used to detect different cytokine levels. Levels of cytokines were quantified by multi-analyte ELISA. The absorption was measured at 450 nm by the spectrophotometer. Absorbance values more than two times the corresponding negative control absorbance values are interpreted as positive samples according to manufacturers’ instructions and indicated with an asterisks (*). rNP: mice immunized with rNP, NC: mice immunized with PBS as a negative control group.

In many studies, the analysis of cytokines produced in response to antigens is used to gauge the nature of the immune response generated against the antigen. In this study, cytokines released from rNP stimulated lymphocytes of rNP immunized mice into the culture medium were assessed to analyze rNP-specific T cell response. The 5.6, 3.25 and 2.1-fold increase for IL-2, IFN-γ, and GM-CSF respectively and 2.2 fold increase for IL-4 was detected compared to negative control used in the commercial EIA test (Fig 7). As expected, the presence of IL-2 and IFN-γ corroborates the observations obtained from DTH and LP assay suggesting Th1 mediated immune response against CCHFV NP. Additionally, 5.4 and 1.8-fold increase was detected for IL-17A and low IL-10, respectively.

## Discussion

In this study, NP was utilized as a tool to assess the induction capacity for both humoral and cellular immunity in multiple hosts. The expression of CCHFV rNP in *E*. *coli* showed that, even though the substantial amount of protein production achieved, expressed proteins accumulated as inclusion bodies. However, successful purification of rNP in denaturing conditions followed by dialysis yielded a properly folded protein.

The native structure of the rNP has been confirmed with CCHFV antibody positive human sera in an EIA assay and this rNP reacted with IgG generated to viral NP. Also, sera from inactivated-CCHFV immunized rabbit and mice reacted with recombinant NP. Together these results indicated that bacterially expressed rNP bears the necessary antigenic structures required for accurate recognition. There are a number of studies using recombinant CCHFV NP produced in different hosts, like plants, bacteria, or insect cell lines, and detecting IgG or IgM antibodies in sera from CCHFV survivors [26,31–34]. In a recently published study, the suitability of recombinant NP for the diagnosis proven once again and bacterially expressed CCHFV rNP could be used successfully for the serosurveillance of different species [35]. Together with the results from these studies, our data confirm that rNP could be an ideal antigen and an excellent surrogate that can substitute the whole virus in diagnostics [31–33,35].

Nucleoprotein of CCHFV is the most abundant protein in viral particles and participates in most processes in CCHFV biology [15]. Formation of ribonucleoprotein structures through interacting with viral genomic and anti-genomic RNA and RNA-dependent RNA polymerase, viral packaging, transcription, and replication are among the cardinal roles of NP [1,16,36–38]. Alongside these roles, NP also interferes with the establishment of the antiviral state in the host cells by interacting with the components of the apoptosis pathways or by suppressing the interferon response [20,22,39]. Not surprisingly, NP is also highly immunogenic, and the main target of B and T cells [17,27,40].

As it has been shown with many other viruses like influenza A and Ebola virus and also viruses in *Bunyavirales* order like Rift Valley Fever Virus, Lassa Virus and Hantaviruses, internal viral proteins with highly conserved sequences, when compared to surface proteins that prone to mutations, are targets for long-lasting immunity [41–47]. By being the most abundant protein in viral particle [16], also one of the main targets of immunity [27], NP has chosen for this study to explore and exploit its priming capacity for both humoral and cellular immunity. Even though the immunization of mice with rNP alone does not represent the natural course of infection and immunity, a high titer of IgG found in immunized mice sera proves that rNP actually stimulates humoral immunity and is recognized by CD4+ cells as a prominent antigen in the mouse. Further exploration of rNP in the generation of humoral immune response with the immunized rabbits revealed NP-specific IgG response. Even though the antibody titer in rabbit sera was not high as it was in mice sera, this result suggests NP as a stimulator of humoral immunity in multiple hosts. Assaying human serum from recovered CCHFV patients and whose positivity for CCHFV specific antibodies was previously confirmed with a commercial test, we were able to detect NP-specific antibodies in positive sera with 89,2% sensitivity and 91,3% specificity (Fig 4). This detection rate, compared to commercial EIA using the inactivated whole virus as a capture antigen, points out the abundance of NP-specific IgG in CCHFV infected humans. The generation of high IgG titers in multispecies is a convincing indicator that NP is an excellent antigen for B cells. It could be argued that antibodies against an internal protein such as NP, may not be effective in neutralizing virus infection and therefore has limited value in inducing sterilizing immunity, However, data from various vaccine studies unequivocally shown that NP serves as an integral part of protective immunity in many vaccine formulations [27,48–50]. It is likely that NP succeeds this by not directly neutralizing the virus itself but by indirect means such as stimulating a vigorous cellular immunity as our data demonstrated in multispecies scrutinies. Among the possible means by which virus infection could be constrained are the cytotoxic T cell activity and DTH type inflammatory responses. Together with the above-mentioned results and other previous studies, it could be stated that there is a strong NP-specific antibody response during the natural course of infection [51,52], and CD4+ cell epitopes on NP are recognized in multispecies.

The central role of CD4+ cells on immunity is not just through the promotion of antibody production by B cells but also by driving the generation of CD8+ T cells. In many viral infections, CD4+ effector cells are mainly Th1 subtypes [53]. Virus specific T cell response was found to be predominantly against epitopes on NP in survivors of CCHFV [27]. For this reason, we wanted to assess the presence and strength of Th1 cell-mediated response to rNP both *in vivo* and *in vitro*. For the *in vivo* demonstration of the cell-mediated immunity, the DTH reaction, which is primarily a Th1 type response [54], was assessed. In mice, the comparison of thickness in rNP injected footpads versus PBS injected controls, showed in a significant difference, indicating strong Th1-mediated DTH response. For the *in vitro* correlate of DTH, the proliferative responses of the lymphocytes from rNP immunized mice, iCCHFV immunized rabbit, and also from CCHFV survivors to rNP antigen were tested. In this method, the induction and magnitude of the cellular response are measured by the level of lymphoproliferation in the presence of the antigen. Significant levels of lymphoproliferative responses were detected both in experimental animals and CCHFV survivors once again leading to the conclusion that NP is a strong inducer of T helper cell activity. The *in vitro* evaluation of cell-mediated response can also provide valuable information regarding the cellular components of the observed reaction. This could be achieved by the determination of the dominating cytokines in the culture supernatants. Therefore, we investigated the level of different cytokines in the test environments. Detailed profiling of cytokines from these wells revealed increased levels of IL-2 and IFN-γ, which are indicators of Th1 mediated immune response. An increase in the IL-4 level and, slight but not significant increase in the IL-10 level indicated that Th2 response was also present in rNP antigen-stimulated lymphocytes providing an explanation for the robust antibody production following rNP immunizations. The cytokine levels in natural course of infection have been investigated in a number of studied and IL-6, IL-10, TNF-α and IFN-γ were found to be major cytokines during the natural course of infection. Among these, IL-6 and TNF-α levels were found to be mostly associated with fatal outcome [55,56]. IL-10 and IFN-γ levels were reported to be high in patient group with fatal outcome [57], yet in another study IFN- γ levels were high both in fatal and non-fatal group [58]. High IL-10 levels were also found to be associated with disease severity among surviving patients [57]. In an immunocompetent mice, NP based CCHFV vaccines developed with different platforms, uniformly induced IL-2, IL-10, IL-6, Il-4, TNF-α and IFN-γ [59]. In a CCHFV NP based mRNA vaccine study, IFN-γ an IL-4 levels were high in vaccinated mice before challenge [60]. The results from our study shows that rNP induced cytokines that could indicate balanced Th1/Th2 response. Another interesting result was the IL-17 level, together with the low IL-10 level, suggesting the activities of Th17 cells. The role of Th17 cells induced by rNP immunizations requires further investigations and warrants the scrutiny of the possible link between NP and the excessive inflammatory response observed in CCHFV infection.

One of the limitations of our study is related to the protein production process. Any presumptive conformational changes during the denaturation and refolding processes might prevent the exposure of NP epitopes to components of the immune system. The corroborating evidence for NP-specific Th1 response should be addressed in the in vivo infection model. The other is about the scope of this study. Here, only the similarities in immune responses in the multiple hosts were investigated. But further experiments to reveal dissimilarities in immune responses of different hosts will unveil important determinants for viral pathogenesis.

Detailed studies such as carried out in this report on adaptive immune response, specifically on the cellular immune response to CCHFV and particularly against individual proteins are rather scarce. Investigation of the roles of each viral protein in adaptive immunity will contribute to the delineation of the full picture on immunology and pathogenesis of CCHFV infection. In this study, recombinant CCHFV NP was used in the investigation of its role in adaptive immunity. Even though during the natural course of infection, there are many other viral/host factors directing the type of effector responses, our results show that rNP alone induces CD4+ T cell response and sufficient to drive this response towards Th1. Further exploration of this priming capacity and function of NP in the generation of different T cell subsets, including memory cells, will extend our knowledge related to protective immunity, which has paramount importance not only in developing a well-rounded vaccine approach but also in understanding the complex pathogenesis of this deadly disease.
